# Association Between Exclusive Breastfeeding, Nutritional Status and Eating Behavior, in Chilean Schoolchildren: A Cross-Sectional Study

**DOI:** 10.3390/nu17213444

**Published:** 2025-10-31

**Authors:** Nicole Lasserre-Laso, Guillermo Leiva-Manzor, Edson Bustos-Arriagada, Karina Etchegaray-Armijo

**Affiliations:** 1 Escuela Nutrición y Dietética, Facultad de Salud, Universidad Santo Tomás, Los Ángeles 4440000, Chile; nlasserre@santotomas.cl (N.L.-L.); gleivam@alumnos.santotomas.cl (G.L.-M.); 2 Faculty of Medicine, Nutrition and Dietetics School, Universidad Finis Terrae, Pedro de Valdivia 1509, Providencia, Santiago 7501015, Chile; edsonbustos@uft.cl

**Keywords:** exclusive breastfeeding, eating behavior, nutritional status, overweight, schoolchildren

## Abstract

**Objectives:** The aim of this study was to analyze the association between exclusive breastfeeding (EBF) up to six months and both nutritional status and eating behavior in Chilean schoolchildren aged 5 to 8 years. **Methods**: A cross-sectional study was conducted involving 109 children aged 8–10 years from three cities in Chile. EBF was assessed retrospectively through caregiver questionnaires. Eating behavior was evaluated using the Spanish version of the CEBQ. Anthropometric measurements (weight, height, and waist circumference) were obtained following a standardized protocol to determine nutritional status and waist-to-height ratio. Differences between EBF and non-EBF groups were analyzed using the Wilcoxon–Mann–Whitney and Kruskal–Wallis tests. **Results:** Eating behavior scores for pro-intake (“food approach”) were strongly associated with obesity among non-EBF children (ratio = 1.24, *p* = 0.001). Children with abdominal obesity who were not EBF showed higher scores for food approach behaviors (ratio = 1.22, *p* = 0.011). A significant trend was observed in the subscale Enjoyment of Food and Emotional Overeating in children with obesity (*p* = 0.011 and *p* = 0.008), abdominal obesity (*p* = 0.031 and *p* = 0.017), and cardiometabolic risk (*p* = 0.036 and *p* = 0.049) in the non-EBF group, compared to those who were EBF up to 6 months of age. **Conclusions:** Scores reflecting a tendency toward higher food intake were strongly associated with obesity and other obesity-related parameters in children who did not experience EBF during early childhood. Having been exclusively breastfed, regardless of current nutritional status, was associated with lower scores on pro-obesity behaviors.

## 1. Introduction

Breastmilk is considered the optimal food for infants, providing a safe, clean source of nutrition containing antibodies that protect against many common childhood illnesses. All the energy and nutrients required by infants during the first months of life are supplied by breastmilk, and it continues to provide at least half of a child’s nutritional requirements during the latter half of the first year, and up to one-third during the second year of life [1]. Nutrition is not the only benefit attributed to breastfeeding (BF); it is also regarded as a core component of parenting that supports biological, psychological, and social development [2]. Adaptation, early behavioral learning, and the formation of essential social bonds are promoted through BF [3].

As the first form of feeding for many infants, BF has been proposed as an early target for interventions aimed at promoting healthier eating behaviors from a young age [4]. Eating behavior is defined as the relationship between humans and food; it is influenced by a complex interplay of genetic and environmental factors, established during childhood [5], and shaped by experiences and learning in the early years of life [6]. The acceptance of new foods during complementary feeding, as well as the development of chemosensory perceptions—particularly in taste and smell—may be influenced by exposure to the variety of flavors present in the maternal diet through BF [7,8]. These effects, mediated by intrauterine and postnatal exposure to maternal dietary flavors, are thought to contribute to greater acceptance of these foods later in life [9,10,11].

Associations between BF and food preferences have been reported in previous research. Longer durations of BF have been shown to be significantly associated with increased variety and quantity of fruits and vegetables consumed in childhood [11,12], independent of sociodemographic factors [13,14]. Exclusive BF (EBF) for up to six months has also been linked to favorable dimensions of eating behavior, as assessed through validated psychometric tools, primarily in preschool and early school-aged children [4,5,15]. Protective eating behaviors—including slower eating, lower desire for sugary foods, and reduced responsiveness to food and emotional overeating—have been reported to be associated with longer durations of BF [5,15,16,17].

It has been previously reported that eating behavior can influence nutritional status. In Spanish schoolchildren, pro-intake subscales (enjoyment of food, food responsiveness, emotional overeating, desire for drinks) were positively related to excess weight by Body Mass Index (BMI) and high adiposity [18,19].

Few studies have been conducted in Chile to examine the association between BF and eating behavior in schoolchildren. Therefore, the aim of this study was to analyze the association between exclusive breastfeeding up to six months and both nutritional status and eating behavior in Chilean schoolchildren aged 5 to 8 years.

## 2. Materials and Methods

### 2.1. Design and Subjects

This was a quantitative, cross-sectional, correlational study. The study population consisted of *n* = 109 schoolchildren aged 5 to 8 years, along with their mothers, from one public school in each of three cities in Chile (Iquique, Los Ángeles, and Temuco) that had an existing agreement with the university. Evaluations were conducted over a six-month period. A non-probability convenience sampling method was used, and participants were selected based on convenience and whether the mother had signed the informed consent form [20]. Inclusion criteria were enrollment in the school during the study period and availability of the child’s health record from the primary care service during the interview. Exclusion criteria included children or mothers with a clinical diagnosis of neurocognitive disorders, such as autism spectrum disorder, which could be associated with specific eating behaviors, as well as any conditions that might affect the child’s nutritional status or dietary intake, including gastrointestinal or metabolic disorders, severe food allergies, or chronic illnesses affecting feeding. Background information was obtained through interviews with each mother, who reported sociodemographic, anthropometric, and breastfeeding variables. All information was reported by the mothers. Anthropometric measurements and the administration of the questionnaire were carried out by trained professionals.

### 2.2. Exclusive Breastfeeding

The duration of BF was obtained retrospectively through maternal recall. Mothers were first asked whether their child had ever been breastfed [21]. Those who responded affirmatively were subsequently asked about the duration of breastfeeding in months, whether it had been exclusive or supplemented with other foods, the age at which infant formula was introduced, and the timing of complementary feeding. Based on these responses, breastfeeding was classified as exclusive (EBF) if it was maintained for six months or longer and no formula or complementary foods were introduced before six months; otherwise, it was classified as non-exclusive (non-EBF) [1].

### 2.3. Anthropometric Measurements

Anthropometric evaluations were conducted to assess the nutritional status of the schoolchildren, including measurements of height, body weight, and waist circumference (WC) following standardized protocols [22]. Height was measured using a SECA^®^ mobile stadiometer (model 217, Hamburg, Germany), and body weight was recorded with a SECA^®^ digital floor scale (model 876, Hamburg, Germany). WC was measured at the midpoint between the lowest rib and the iliac crest using a SECA^®^ ergonomic measuring tape (model 201, Hamburg, Germany), with the tape positioned horizontally and snugly around the waist without compressing the skin.

Body mass index (BMI) was calculated to determine BMI-for-age and classify the nutritional status of the schoolchildren. WC was classified using the WC-for-age (WC/A) indicator, based on WHO growth standards adapted by the Chilean Ministry of Health [23]. The waist-to-height ratio (WHtR) was calculated to evaluate central obesity and cardiometabolic risk, using the evidence-based cutoff of ≥0.5 [24].

### 2.4. Eating Behavior Assessment

Eating behavior was assessed using the Child Eating Behavior Questionnaire (CEBQ), which was administered to mothers of the schoolchildren by trained interviewers. The CEBQ is a validated instrument developed to evaluate eating patterns in children [25]. The questionnaire was adapted to Chilean Spanish in 2011, following a forward–backward translation protocol and cultural adaptation through cognitive interviews with ten mother–child dyads [26,27].

The CEBQ includes 35 items grouped into eight subscales that assess different aspects of eating behavior. Mothers rated their child’s eating habits on a five-point Likert scale ranging from 1 (“Never”) to 5 (“Always”). This instrument includes eight subscales, organized into two main dimensions: Food Approach and Food Avoidant.

The Food Approach dimension comprises the subscales: Enjoyment of Food (4 items), Food Responsiveness (5 items), Emotional Overeating (4 items), and Desire to Drink (3 items) [26,27]. The first two subscales reflect a greater attraction to food, associated with eating enjoyment and increased responsiveness to food-related stimuli, while Emotional Overeating captures the tendency to increase food intake in response to negative emotional states, and Desire to Drink reflects the frequent desire to consume beverages, particularly sugary drinks [6].

The Food Avoidant dimension includes Satiety Responsiveness (5 items), Slowness in Eating (4 items), Emotional Undereating (4 items) and Food Fussiness (6 items). These subscales indicate lower motivation toward food intake, characterized by greater sensitivity to satiety and a slower pace of eating. Emotional Undereating assesses the tendency to reduce food intake during negative emotional states, whereas Food Fussiness captures food selectivity or reluctance to accept a wide variety of foods [25,26,28].

### 2.5. Statistical Analysis

Categorical variables were expressed as absolute and relative frequencies. Continuous variables were assessed for normality using the Shapiro–Wilk test. Normally distributed variables were reported as mean and standard deviation (SD), while non-normally distributed variables were reported as median and interquartile range (IQR).

Raw scores for each CEBQ subscale were obtained by summing the item responses. To standardize the results, each subscale score was divided by the number of items, producing values on a 1-to-5 scale. A behavioral ratio was also calculated, with ratios greater than 1 indicating a predominance of food approach tendencies and ratios less than 1 indicating a predominance of food avoidant tendencies.

Radar plots were generated to visualize CEBQ subscale scores, with Food Approach behaviors displayed on the right and Food Avoidant behaviors on the left. Children who received EBF were depicted in blue, while non-EBF children were depicted in red.

Associations between BF status and eating behavior, as well as between eating behavior and WHtR, were analyzed using the Wilcoxon–Mann–Whitney U test. Associations between eating behavior and BF status stratified by nutritional status and WC were analyzed with the Kruskal–Wallis test, followed by post hoc comparisons to identify significant group differences.

All analyses were conducted using Stata software, version 15.1 (StataCorp LLC, College Station, TX, USA). A *p*-value < 0.05 was considered statistically significant.

### 2.6. Ethical Considerations

The project and its documentation were approved by the Scientific Ethics Committee of the University of Santo Tomás and the Scientific Ethics Committee of the Biobío Health Service. All participants signed an informed consent form confirming their willingness to be included in the research. Additionally, the schoolchildren signed an age-appropriate informed assent form, indicating their agreement to participate in the study.

## 3. Results

Table 1 presents the sociodemographic, anthropometric, and BF characteristics of the study participants. The mean age of the children was reported as six years. Anthropometric measurements showed that 58% of the children were classified as having overweight or obesity, and 18.3% were classified as having abdominal obesity. Additionally, 51.4% were identified as being at cardiometabolic risk according to the waist-to-height ratio (WHtR). Regarding exclusive breastfeeding (EBF), 50.5% of the participants reported having received EBF, with a mean duration of 17.4 months.

**Figure 1:**
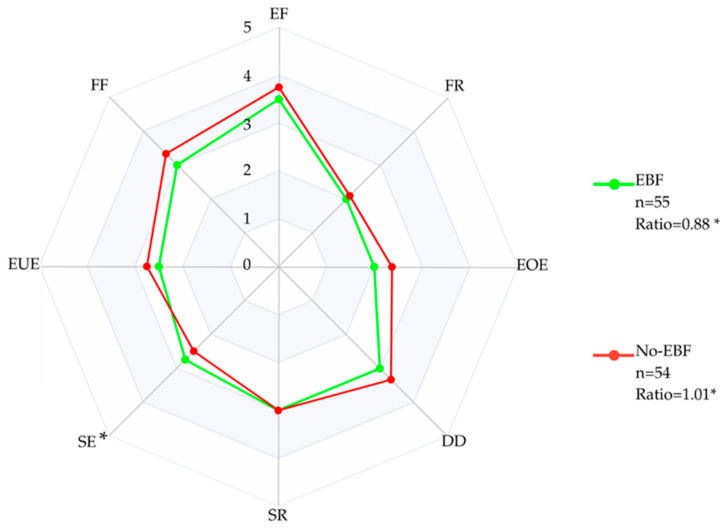
Median scores of eating behavior among school-aged children by Exclusive Breastfeeding. Exclusively breastfed (EBF, green) versus those who were not (non-EBF, red). On the right side, the subscales reflecting a tendency toward increased food intake (“Food Approach”: Enjoyment of Food [EF], Food Responsiveness [FR], Emotional Overeating [EOE], and Desire to Drink [DD]). On the left side, the subscales reflecting food avoidance behavior (“Food Avoidant”: Satiety Responsiveness [SR], Slowness in Eating [SE], Emotional Undereating [EUE], and Food Fussiness [FF]). (* *p* = 0.03).

Figure 1 presents radar charts illustrating the median scores of eating behavior among school-aged children who were classified as exclusively breastfed (EBF, in green line) versus those who were classified as non-exclusively breastfed (non-EBF, in red line). Significant differences were found in Slowness in Eating (*p* = 0.04), with EBF children being observed to have higher scores than non-EBF children, as well as a greater tendency toward increased intake observed among non-EBF children compared to EBF children (ratio = 1.01, *p* = 0.03).

Eating behavior scores within the “Food Approach” dimension, which are associated with greater food intake, were found to be strongly associated with obesity among non-EBF children (ratio = 1.24, *p* = 0.001) (Table 2), particularly in relation to the Enjoyment of Food, Food Responsiveness, and Emotional Overeating subscales (*p* = 0.001 for all). Conversely, within the “Food Avoidant” subscales, Slowness in Eating was observed to be significantly and inversely associated with obesity in non-EBF children (*p* = 0.03).

Similarly, a positive association was observed between the “Food Approach” subscale and waist circumference (Table 3). Higher scores for food approach behaviors were reported in non-EBF children with abdominal obesity compared to those with normal WC or at risk of abdominal obesity (ratio = 1.22, *p* = 0.011). Specifically, elevated scores were observed in the subscales of Enjoyment of Food, Food Responsiveness, and Emotional Overeating (*p* = 0.001, *p* = 0.008, and *p* = 0.005, respectively).

When the WHtR was examined (Table 4), a positive association was also identified with “Food Approach” subscales, particularly the subscales of Enjoyment of Food, Food Responsiveness, and Emotional Overeating (*p* = 0.020, *p* = 0.003, and *p* = 0.009, respectively) among children at cardiometabolic risk who were non-EBF. In contrast, higher scores in the “Food Avoidant” subscales, specifically Satiety Responsiveness and Slowness in Eating (*p* = 0.013 and *p* = 0.001), were reported among EBF children without cardiometabolic risk.

A significant trend was also noted toward higher scores in the “Food Approach” subscales, specifically Enjoyment of Food and Emotional Overeating, in children with obesity (*p* = 0.011 and *p* = 0.008), abdominal obesity (*p* = 0.031 and *p* = 0.017), and cardiometabolic risk (*p* = 0.036 and *p* = 0.049) compared to those who had been EBF for up to 6 months. Additionally, an inverse trend was observed in the “Food Avoidant” subscale Food Fussiness, with higher scores among EBF children with obesity (*p* = 0.009), abdominal obesity (*p* = 0.017), and cardiometabolic risk (*p* = 0.017) (Table 5).

## 4. Discussion

The results of this study indicated that obesity was significantly associated with higher scores in eating behavior dimensions, reflecting a greater inclination toward increased food intake. Among children who had not been exclusively breastfed and presented obesity, abdominal obesity, or cardiometabolic risk, significantly higher scores were observed in the CEBQ subscales of Enjoyment of Food, Food Responsiveness, and Emotional Overeating, compared to children with normal nutritional status. Conversely, exclusive breastfeeding was associated with healthier eating behavior patterns, even in children with adverse nutritional profiles [29,30]. These findings are particularly relevant, as they suggest that school-aged children with obesity and cardiometabolic risk tend to exhibit eating behaviors characterized by increased energy intake. Moreover, they indicate that EBF may act as a protective factor, modulating these behaviors even in children who are already overweight or obese.

Eating behavior, as measured by the CEBQ, has been described as a relatively stable trait over time, with strong validity and internal consistency [31,32,33]. Several studies have reported positive associations between Food Approach subscales and BMI in children [27,29]. Consistent with our findings, previous research has shown that children with obesity exhibited higher scores in Enjoyment of Food and Food Responsiveness, reflecting greater attraction to food and increased sensitivity to external food cues, as well as higher Emotional Overeating scores, indicating a tendency to eat in response to negative emotional states [27,34]. Additionally, variables such as Satiety Responsiveness and Emotional Overeating have been positively linked to BMI in children [35,36].

However, few studies have assessed the association between CEBQ scores and WHtR, despite WHtR being recognized as a more sensitive marker of cardiometabolic risk in childhood than BMI or WC alone [29,37,38,39]. A recent study in Spanish schoolchildren reported a positive association between WHtR and all Food Approach subscales of the CEBQ [18] while another cross-sectional study found that fast eating—an observable eating behavior—was associated with WHtR ≥ 0.5 [30,40]. These findings support the hypothesis that central adiposity, as reflected by elevated WHtR, may be influenced by specific eating behavior patterns.

Delayed satiety, often observed in children with overweight, has been linked to dysregulation in appetite control, increased gastric capacity, and heightened responsiveness to external cues such as high-calorie and palatable foods [41,42].

BF has been described as a protective factor against childhood and adolescent obesity, and a lower risk of metabolic alterations [1,43]. In this regard, the first 1000 days of life (from gestation through 24 months) have been identified as a critical window for development, prevention, and the establishment of healthy eating habits [7]. BF in the postpartum stage stands out as one of the main protective factors against childhood obesity, associated with various mechanisms including the nutritional composition of human milk, the contribution of bioactive substances such as hormones and immunological factors, and the modulation of energy intake regulation and eating behavior development [8,44].

One hypothesis proposed by the literature is that exposure to diverse flavors through BF, derived from the maternal diet, can influence the acceptance of new foods during complementary feeding and the development of sensory experiences, particularly taste and smell [7,8]. This exposure, initiated during intrauterine life and continued through BF, could promote long-term acceptance of varied foods during complementary feeding [10,11,45].

A cohort study conducted in Brazil found that breastfeeding duration is associated with lower consumption of ultra-processed foods and higher consumption of fresh foods during childhood, adolescence, and adulthood, indicating a long-term influence on eating habits [46]. In this context, a systematic review that included seven studies evaluating the influence of breastfeeding duration on eating behavior in children aged 2 to 6 years reported results consistent with those of the present study [47]. Most of the included studies demonstrated a positive association between longer breastfeeding duration and healthier eating behaviors [16], including reduced food neophobia, lower “food responsiveness” [14] and decreased “fussy eating” behaviors in children within this age group [48].

Schoolchildren who were not exclusively breastfed were found to exhibit a tendency toward “pro-intake” behaviors, supporting the hypothesis that exclusive BF may have a modulatory effect on appetite self-regulation. This is consistent with studies suggesting that exclusive BF promotes energy self-regulation during childhood [48,49].

### Strengths and Limitations

One of the main strengths of this study is considered to be the use of the CEBQ, a widely validated instrument that has been culturally adapted for the Chilean context, which supports the reliability of the eating behavior assessments. Additionally, the inclusion of a comprehensive evaluation of nutritional status—through measurements of BMI, WC, and WHtR-allowed for a more complete characterization of nutritional risk.

However, this study is also associated with several limitations. Its cross-sectional design does not allow causal inferences between EBF, eating behavior, and obesity. The reliance on self-reported questionnaires and retrospective reporting of EBF may introduce recall bias or social desirability. Moreover, potential confounding factors such as family environment and socioeconomic status were not controlled for.

It is also important to recognize that both BF and child eating behavior are complex processes. Therefore, it is challenging to attribute the protective effect of BF to a single mechanism. Furthermore, obesity is recognized as a multifactorial disease, influenced by maternal, child, cultural, genetic, and environmental factors [50]. Several potential confounders in establishing between BF and obesity have been described, such as pregestational and gestational history, and maternal nutritional status [51], variations in underlying genetic traits, interindividual differences in the infant’s metabolome regulating appetite, food intake, or energy expenditure, and methodological limitations of existing studies [44]. In practice, the combined interactions among these components are inseparable and exert a dynamic effect on the child’s eating experience and nutritional status. The complexity presents a challenge for future research, requiring longitudinal designs and causal model to establish new mechanisms and elucidate their interactions both globally and within Chile.

## 5. Conclusions

BF is considered a natural, cost-effective practice with multiple benefits, including protection against childhood obesity, making it a promising strategy for the early prevention of obesity, which is a global epidemic. The results of this study indicate that children who had been EBF, regardless of current nutritional status, were associated with lower scores in pro-obesity eating behaviors.

However, BF and the development of eating behavior are recognized as dynamic processes that interact with multiple factors. Therefore, future research with higher methodological quality is required to demonstrate the association between BF and eating behavior in infancy. Only then will clearer causal relationships be established, generating recommendations based on robust and comprehensive evidence that address the complex interplay of biological, environmental, and cultural factors.

It remains essential to continue promoting EBF until 6 months of age and to continue BF supplemented with semi-solid foods until at least the first year of life, given its well-documented benefits and its association with longer-term positive feeding outcomes.

## Figures and Tables

**Table 1:** Characteristics of study participants: sociodemographic, anthropometric, and breastfeeding variables. X = average, SD = standard deviation.

Sociodemographic Characteristics		
	*n*	%
City of residence		
Los Ángeles	61	56.0
Temuco	18	16.5
Iquique	30	27.5
Gender		
Female	58	53.2
Male	51	46.8
Anthropometric Characteristics		
Weight (kg) (X ± SD)	26.22	5.70
Height (cm) (X ± SD)	119.94	6.69
BMI (kg/m^2^) (X ± SD)	18.06	2.61
Waist circumference (cm) (X ± SD)	59.87	7.74
Waist-to-height ratio (X ± SD)	0.49	0.05
Nutritional Status		
Normal	45	41.7
Overweight	39	36.1
Obese	24	22.2
Waist circumference classification		
Normal	55	50.5
Risk of abdominal obesity	34	31.2
Abdominal obesity	20	18.3
Waist-to-Height Ratio Classification		
Normal	53	48.6
Cardiometabolic risk	56	51.4
Breastfeeding Characteristics		
Duration of breastfeeding (months) (X ± SD)	17.40	12.62
Exclusive Breastfeeding		
EBF	55	50.5
Non-EBP	54	49.5
Age of introduction to infant formula (months) (X ± SD)	10.60	9.94
Age of introduction to complementary feeding (months) (X ± SD)	5.82	0.61

**Table 2:** Association of the dimensions of eating behavior with nutritional status (BMI) according to exclusive breastfeeding. EBF = exclusive breastfeeding. Non-EBF: non-exclusive breastfeeding. N = normal nutritional status (according to BMI by age). OW = overweight. OB = Obesity. Kruskal–Wallis test and post hoc. *p*-value * < 0.05; Different superscript letters (^a,b,c^) indicate statistically significant differences between groups after post hoc analysis (*p* < 0.05).

	EBF				Non-EBF			
	N *n* = 24	OW *n* = 21	OB *n* = 9	*p*-Value	N *n* = 21	OW *n* = 18	OB *n* = 15	*p*-Value
Enjoyment of food	3.25 [2.87–3.75]	3.5 [2.75–4.0]	3.5 [2.75–4.0]	0.78	3.5 [2.75–4.0] ^a^	3.5 [3.0–4.0] ^a,b^	4.25 [4.0–4.75] ^c^	0.00 *
Food responsiveness	1.7 [1.4–2.2]	2.0 [1.2–2.6]	2.0 [1.6–3.0]	0.77	1.4 [1.0–2.0] ^a^	2.1 [1.2–2.6] ^a,b^	3.2 [2.4–3.4] ^c^	0.00 *
Emotional over-eating	1.87 [1.25–2.25]	2.25 [1.5–3.25]	2.0 [1.5–2.75]	0.18	1.5 [1.0–2.5] ^a^	2.37 [1.25–3.0] ^a,b^	3.25 [2.75–3.75] ^c^	0.00 *
Desire to drink	3.0 [2.0–4.0]	2.33 [1.66–4.0]	4.0 [2.0–4.0]	0.64	3.0 [2.0–4.0]	4.0 [3.0–4.6]	3.33 [2.0–5.0]	0.38
Satiety responsiveness	3.1 [2.9–3.6] ^a^	2.8 [2.6–3.4] ^a,b^	2.4 [2.2–2.8] ^b^	0.00 *	3.0 [2.6–3.6]	2.8 [2.4–3.2]	2.8 [2.2–3.2]	0.28
Slowness in eating	3.0 [2.62–4.25]	2.75 [2.0–3.75]	2.5 [2.5–3.0]	0.14	3.0 [2.25–4.0] ^a^	2.5 [2.0–3.5] ^a,b^	2.5 [1.75–2.5] ^b^	0.03 *
Emotional under-eating	2.75 [2.0–3.0]	2.75 [2.0–3.0]	2.25 [2.0–2.75]	0.48	2.75 [2.0–3.0]	2.75 [2.25–3.25]	2.5 [1.5–3.25]	0.65
Food fussiness	2.83 [2.66–3.5]	3.0 [2.5–4.16]	3.0 [2.33–3.16]	0.66	3.33 [2.83–3.83]	3.0 [2.6–3.5]	3.5 [3.16–4.16]	0.09
Ratio	0.79 [0.65–0.95] ^a^	0.9 [0.81–1.06] ^a,b^	1.0 [0.91–1.13] ^b^	0.02 *	0.91 [0.74–1.01] ^a^	1.02 [0.78–1.18] ^a,b^	1.24 [1.03–1.47] ^b^	0.00 *

**Table 3:** Association of the dimensions of eating behavior with waist circumference status according to exclusive breastfeeding. N = normal waist circumference. RAO = risk of abdominal obesity according to waist circumference. AO = abdominal obesity. Kruskal–Wallis test and post hoc, *p*-value * < 0.05; Different superscript letters (^a,b^) indicate statistically significant differences between groups after post hoc analysis (*p* < 0.05).

	EBF				Non-EBF			
	N *n* = 29	RAO *n* = 18	AO *n* = 8	*p*-Value	N *n* = 26	RAO *n* = 16	AO *n* = 12	*p*-Value
Enjoyment of food	3.25 [3.0–3.5]	3.75 [3.0–4.0]	3.25 [2.62–4.25]	0.526	3.37 [2.75–4.0] ^a^	3.87 [3.37–4.37] ^a,b^	4.25 [4.0–4.62] ^b^	0.001 *
Food responsiveness	1.6 [1.4–2.0]	2.5 [1.2–3.0]	2.0 [1.4–3.0]	0.410	1.5 [1.0–2.6] ^a^	2.2 [1.2–3.2] ^a,b^	3.2 [2.4–3.4] ^b^	0.008 *
Emotional over-eating	2.0 [1.25–2.25]	2.75 [1.25–3.25]	2.12 [1.5–2.75]	0.174	1.62 [1.25–2.5] ^a^	3.0 [1.25–3.25] ^a,b^	3.12 [2.5–3.37] ^b^	0.005 *
Desire to drink	3.0 [2.0–4.0]	3.3 [2.0–4.6]	2.0 [1.5–4.16]	0.507	3.5 [2.0–4.66]	3.83 [3.0–4.33]	3.0 [2.0–3.3]	0.387
Satiety responsiveness	3.0 [2.8–3.6] ^a^	2.9 [2.6–3.4] ^a,b^	2.4 [2.2–2.7] ^b^	0.014 *	3.0 [2.6–3.4]	2.8 [2.4–3.2]	2.9 [2.3–3.2]	0.577
Slowness in eating	3.0 [2.75–4.25]	2.5 [2.0–4.0]	2.62 [2.25–3.12]	0.127	2.87 [2.25–4.0] ^a^	2.5 [2.0–3.0] ^a,b^	2.25 [1.75–2.5] ^b^	0.020 *
Emotional under-eating	2.75 [2.0–3.0]	2.6 [2.0–3.2]	2.25 [2.12–2.62]	0.487	2.75 [2.0–3.0]	2.62 [2.0–3.12]	2.75 [2.12–3.25]	0.891
Food fussiness	3.0 [2.6–3.6]	2.9 [2.5–4.1	2.75 [2.25–3.25]	0.435	3.08 [2.66–3.66]	3.33 [3.08–3.66]	3.41 [3.16–4.0]	0.373
Ratio	0.80 [0.70–0.95]	0.95 [0.84–0.95]	0.96 [0.78–1.15]	0.059	0.91 [0.74–1.02] ^a^	1.12 [0.83–1.33] ^a,b^	1.22 [1.00–1.29] ^b^	0.011 *

**Table 4:** Association of the dimensions of eating behavior with waist-to-height ratio (WHtR) according to exclusive breastfeeding. EBF = exclusive breastfeeding. Non-EBF: non-exclusive breastfeeding. No-CR = without or non-cardiometabolic risk according to waist-to-height ratio. CR = cardiometabolic risk according to waist-to-height ratio. Kruskal–Wallis test and post hoc. * *p*-value: <0.05.

	EBF			Non-EBF		
	No-CR *n* = 27	CR *n* = 28	*p*-Value	No-CR *n* = 29	CR *n* = 25	*p*-Value
Enjoyment of food	3.25 [2.75–3.75]	3.63 [2.75–4.0]	0.253	3.5 [2.75–4.0]	4.0 [3.75–4.25]	0.020 *
Food responsiveness	1.8 [1.2–2.2]	2.0 [1.2–2.9]	0.365	1.4 [1.0–2.6]	2.6 [2.2–3.2]	0.003 *
Emotional over-eating	2.0 [1.25–2.25]	2.25 [1.37–3.0]	0.152	1.75 [1.25–2.75]	3.0 [2.25–3.25]	0.009 *
Desire to drink	3.0 [2.0–4.0]	2.33 [1.5–4.3]	0.330	4.0 [2.0–2.4]	3.33 [2.33–4.33]	0.800
Satiety responsiveness	3.0 [2.8–3.6]	2.8 [2.4–3.2]	0.013 *	3.0 [2.6–3.4]	2.8 [2.6–3.2]	0.373
Slowness in eating	3.5 [2.75–4.5]	2.5 [2.12–3.12]	0.001 *	3.0 [2.25–4.0]	2.5 [2.0–2.5]	0.013 *
Emotional under-eating	2.5 [2.0–3.0]	2.62 [2.12–3.0]	0.609	2.75 [2.0–3.0]	2.75 [2.0–3.25]	0.800
Food fussiness	3.16 [2.66–4.0]	2.75 [2.33–3.25]	0.074	3.16 [2.66–3.66]	3.33 [3.16–3.66]	0.316
Ratio	0.82 [0.60–1.01]	0.94 [0.78–1.13]	0.041 *	0.91 [0.76–1.11]	1.13 [0.98–1.30]	0.009 *

**Table 5:** Association of eating behavior dimensions according to obesity, abdominal obesity and cardiometabolic risk in exclusive or non-exclusive breastfeeding. OB = Obesity (BMI by age). AO = abdominal obesity (waist circumference). CR = cardiometabolic risk according to waist-to-height ratio. EBF = exclusive breastfeeding. Non-EBF: non-exclusive breastfeeding. Kruskal–Wallis test and post hoc. * *p*-value: <0.05.

	OB			AO			CR		
	EBF	Non-EBF	*p*-Value	EBF	Non-EBF	*p*-Value	EBF	Non-EBF	*p*-Value
Enjoyment of food	3.5 [2.75–4.0]	4.25 [4.0–4.75]	0.011 *	3.25 [2.62–4.25]	4.25 [4.0–4.62]	0.031 *	3.63 [2.75–4.0]	4.0 [3.75–4.25]	0.036 *
Food responsiveness	2.0 [1.6–3.0]	3.2 [2.4–3.4]	0.067	2.0 [1.4–3.0]	3.2 [2.4–3.4]	0.111	2.0 [1.2–2.9]	2.6 [2.2–3.2]	0.091
Emotional over-eating	2.0 [1.5–2.75]	3.25 [2.75–3.75]	0.008 *	2.12 [1.5–2.75]	3.12 [2.5–3.37]	0.017 *	2.25 [1.37–3.0]	3.0 [2.25–3.25]	0.049 *
Desire to drink	4.0 [2.0–4.0]	3.33 [2.0–5.0]	0.880	2.0 [1.5–4.16]	3.0 [2.0–3.3]	0.433	2.33 [1.5–4.3]	3.33 [2.33–4.33]	0.130
Satiety responsiveness	2.4 [2.2–2.8]	2.8 [2.2–3.2]	0.367	2.4 [2.2–2.7]	2.9 [2.3–3.2]	0.295	2.8 [2.4–3.2]	2.8 [2.6–3.2]	0.886
Slowness in eating	2.5 [2.5–3.0]	2.5 [1.75–2.5]	0.068	2.62 [2.25–3.12]	2.25 [1.75–2.5]	0.077	2.5 [2.12–3.12]	2.5 [2.0–2.5]	0.222
Emotional under-eating	2.25 [2.0–2.75]	2.5 [1.5–3.25]	0.810	2.25 [2.12–2.62]	2.75 [2.12–3.25]	0.243	2.62 [2.12–3.0]	2.75 [2.0–3.25]	0.726
Food fussiness	3.0 [2.33–3.16]	3.5 [3.16–4.16]	0.009 *	2.75 [2.25–3.25]	3.41 [3.16–4.0]	0.017 *	2.75 [2.33–3.25]	3.33 [3.16–3.66]	0.017 *
Ratio	1.0 [0.91–1.13]	1.24 [1.03–1.47]	0.052	0.96 [0.78–1.15]	1.22 [1.00–1.29]	0.105	0.94 [0.78–1.13]	1.13 [0.98–1.30]	0.038 *

## Data Availability

The data that support the findings of this study are available from the corresponding author upon reasonable request.
